# Conjugation of Triterpenic Acids of Ursane and Oleanane Types with Mitochondria-Targeting Cation F16 Synergistically Enhanced Their Cytotoxicity against Tumor Cells

**DOI:** 10.3390/membranes13060563

**Published:** 2023-05-30

**Authors:** Mikhail V. Dubinin, Darya A. Nedopekina, Anna I. Ilzorkina, Alena A. Semenova, Vyacheslav A. Sharapov, Eldar V. Davletshin, Natalia V. Mikina, Yuri P. Belsky, Anna Yu. Spivak, Vladimir S. Akatov, Natalia V. Belosludtseva, Jiankang Liu, Konstantin N. Belosludtsev

**Affiliations:** 1 Department of Biochemistry, Cell Biology and Microbiology, Mari State University, pl. Lenina 1, Yoshkar-Ola 424001, Russia; sem_al.ru@mail.ru (A.A.S.); slav.sharapov@yandex.ru (V.A.S.); natasamikina@gmail.com (N.V.M.); natago_imagination@rambler.ru (N.V.B.); bekonik@gmail.com (K.N.B.); 2 Institute of Petrochemistry and Catalysis, Russian Academy of Sciences, Prospekt Oktyabrya 141, Ufa 450075, Russia; rawbe2007@mail.ru (D.A.N.); eldarik1996@mail.ru (E.V.D.); spivak.ink@gmail.com (A.Y.S.); 3 Institute of Theoretical and Experimental Biophysics, Russian Academy of Sciences, Institutskaya 3, Pushchino 142290, Russia; ilzorkinaanna1998@mail.ru (A.I.I.); akatov.vladimir@gmail.com (V.S.A.); 4 Centre of Preclinical Translational Research, Almazov National Medical Research Centre, St. Petersburg 197371, Russia; belsky59@mail.ru; 5 School of Health and Life Sciences, University of Health and Rehabilitation Sciences, Qingdao 266071, China; j.liu@mail.xjtu.edu.cn

**Keywords:** F16, triterpenic acids, cancer, cytotoxicity, mitochondria, mitocans, oxidative phosphorylation, ROS

## Abstract

The present work shows the cytotoxic effects of novel conjugates of ursolic, oleanolic, maslinic, and corosolic acids with the penetrating cation F16 on cancer cells (lung adenocarcinoma A549 and H1299, breast cancer cell lines MCF-7 and BT474) and non-tumor human fibroblasts. It has been established that the conjugates have a significantly enhanced toxicity against tumor-derived cells compared to native acids and also demonstrate selectivity to some cancer cells. The toxic effect of the conjugates is shown to be due to ROS hyperproduction in cells, induced by the effect on mitochondria. The conjugates caused dysfunction of isolated rat liver mitochondria and, in particular, a decrease in the efficiency of oxidative phosphorylation, a decrease in the membrane potential, and also an overproduction of ROS by organelles. The paper discusses how the membranotropic- and mitochondria-targeted effects of the conjugates may be related to their toxic effects.

## 1. Introduction

**Figure 1:**
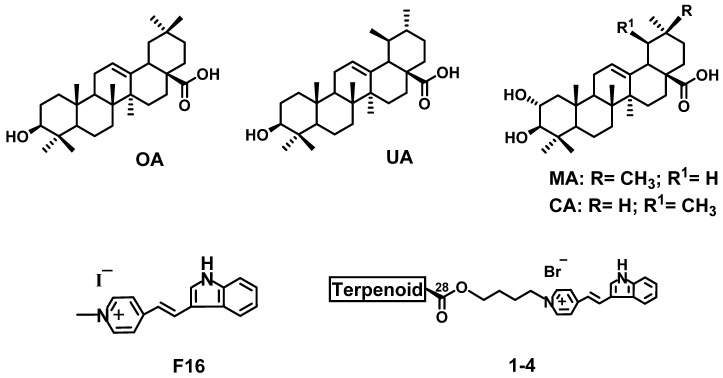
Molecular structures of oleanolic acid (OA), ursolic acid (UA), maslinic acid (MA), corosolic acid (CA), F16 ([E-4-(1H-indol-3-ylvinyl)-N-methylpyridinium iodide]) and of F16-triterpenoid conjugates (**1**–**4**).

Natural pentacyclic triterpenic acids of the ursane and oleanane series, including oleanolic, ursolic, maslinic, and corosolic acids, are widely found in the plant kingdom (Figure 1) [1,2]. These secondary plant metabolites exhibit a diverse range of biological properties, with their cytotoxic activity and capacity to initiate the mitochondrial pathway of apoptosis in various tumor cells being of particular interest [3,4,5,6,7,8,9,10,11,12]. For instance, oleanolic acid induces apoptosis in non-small lung cancer cell lines, even in the presence of anti-apoptotic Bcl-2 proteins, and reduces metastasis in vivo in the B16F10 melanoma model [7]. Ursolic acid triggers autophagy, cell cycle arrest, and apoptosis in different cancer cell lines through multiple signaling pathways, such as NF-kB, STAT3, and TRAIL [5].

Numerous preclinical studies have confirmed the therapeutic potential of maslinic acid and its semi-synthetic derivatives in various tumor types, demonstrating this triterpenoid’s ability to inhibit proliferation, stimulate apoptosis, and obstruct cancer cell angiogenesis [8,9]. Corosolic acid effectively triggers apoptosis in HeLa cervical adenocarcinoma and human prostate cancer (PC-3 and DU145 lines) while exhibiting low toxicity to normal cells and tissues [10,11]. However, the relatively low antitumor potential and high hydrophobicity of triterpenic acids pose significant limitations for their clinical application.

Currently, one promising strategy to address the bioavailability issues and enhance the antitumor action of pentacyclic triterpenoids involves transforming them into derivatives with terminal cationic mitochondrial-targeted groups in the C-3 and/or C-28 side chains. Studies have shown that lipophilic delocalized cations (like TPP^+^ or rhodamine B) facilitate the transmembrane transport of natural triterpenic compounds and boost their mitochondrial targeting [13,14,15,16,17,18,19,20]. Recently, we synthesized a small library of triterpenic acid conjugates with the mitochondria-tropic compound F16 (4-(1-H-indol-3-ylvinyl)-N-methylpyridinium iodide) [21], a small polycyclic cationic molecule known to selectively accumulate in the mitochondrial matrix of various tumor cell lines [22]. The hybrid compounds we obtained demonstrated a significant synergistic enhancement of the toxic effect on human leukemia cells (Jurkat, U937, and K562) with acceptable selectivity for the non-malignant human fibroblast cell line (HPDF). A significant toxic effect of chemically binding triterpenic acids with F16 was also confirmed by studying one of the most active conjugates of betulinic acid and F16 with a triethylene glycol spacer on the MCF-7 breast adenocarcinoma cell line [23]. Confocal fluorescence visualization revealed the high potential of the investigated F16-derived betulinic acid to accumulate in mitochondria. However, we did not observe significant differences in the cytotoxicity of the conjugate between MCF-7 cancer cells and healthy fibroblasts.

The cytotoxicity and selectivity of the cationic derivatives of triterpenic acids may significantly depend on the molecular structure of the triterpenic scaffold, spacer, and type of cationic fragment. Current knowledge about the antitumor effects of natural oxygenated triterpenoids indicates that the configuration and number of hydroxyl groups in ring A of the triterpenic core influence the cytotoxic activity and selectivity of action concerning targets in cancerous and healthy cells [24,25,26]. The molecular structure of oleanolic and maslinic acids or ursolic and corosolic acids differs in the presence of an additional hydroxyl group at the 2-α position in maslinic and corosolic acids. In light of this, we were interested in synthesizing new F16-conjugates with maslinic and corosolic acids and investigating their cytotoxic action compared to the cytotoxic effect of F16-derived ursolic and oleanolic acids previously obtained [21]. We conducted a cytotoxicity analysis using four tumor cell lines (lung adenocarcinoma A549 and H1299, breast cancer cell lines MCF-7 and BT474) and non-tumor human fibroblasts (HPDF). Moreover, we evaluated the mechanism of action of the new derivatives and demonstrated that their cytotoxicity is attributed to a ROS burst. This effect is presumably due to the mitochondrial-targeted action of the compounds and the suppression of mitochondrial function. Indeed, using isolated rat liver mitochondria, we discovered the new compound’s ability to reduce oxidative phosphorylation efficiency and dissipate membrane potential. This may be due to both the protonophore action of the compounds and their capacity to suppress respiratory chain complexes’ activity, which also contributes to the increase in ROS production by mitochondria.

## 2. Materials and Methods

### 2.1. General Procedures

IR spectra (thin films) were obtained with use of a Vertex 70v spectrometer (Bruker, Karlsruhe, Germany). ^1^H and ^13^C NMR spectra were recorded in CDCl_3_ or in MeOD with Me_4_Si as the internal standard on an AVANCE-500 instrument (500.13 (^1^H), 125.78 MHz (^13^C)) or on an AVANCE-400 (400.13 (^1^H), 100.62 MHz (^13^C)) (Bruker, Karlsruhe, Germany). Mass spectra of new compounds were recorded with a Bruker maXis impact high resolution quadrupole time-of-flight mass spectrometer (Bruker, Carteret, NJ, USA). Optical rotation was determined on a 141 polarimeter (Perkin–Elmer, Beaconsfield, UK). Specific rotation [α]_D_ is expressed in (deg·mL)/(g·dm)^−1^; the concentration of the solution *c* is expressed in g/100 mL. Elemental analysis was carried out on a 1106 analyzer (Carlo Erba, Milan, Italy). Thin layer chromatography (TLC) was carried out on Sorbfil plates (Sorbpolimer, Krasnodar, Russia) in chloroform–methanol. Spots were visualized with anisaldehyde. Silica gel L (KSKG grade, 50–160 μm) was employed for column chromatography. All reagents and solvents were of the purest grade available and were generally used without further treatment. The starting compounds ursolic, oleanolic acids, and reagents were purchased from Acros Organics (Geel, Belgium).

### 2.2. Synthesis of F16-Triterpenoid Conjugates

The maslinic and corosolic acids were prepared according to the procedures described in the literature [27]. Physicochemical and spectral characteristics of semisynthetic maslinic and corosolic acids and their bromides 5 and 6 corresponded to the literature data [27,28].

#### 2.2.1. General Procedure for the Preparation of Alkyl Bromides **5** and **6**

1,4-Dibromobutane (5 mmol) was added to a stirred suspension of a maslinic or corosolic acids (0.5 mmol) in waterless DMF (3 mL), MeCN (1 mL), and anhydrous K_2_CO_3_ (0.5 mmol), and the mixture was kept at 50 °C for 3 h. When TLC analyzed that triterpenoid acid was consumed completely, the reaction was quenched with H_2_O and extracted with EtOAc. Anhydrous Na_2_SO_4_ was added to the upper layer to absorb H_2_O, then filtrated and evaporated until it was dry. The residue was purified by column chromatography on silica gel to obtain alkyl bromide as a white flake solid. The yield was 66–74%.

4-Bromobutyl-2α,3β-dihydroxy-olean-12-en-28-oate (**5**). Spectral data and all constants for that compound were as previously reported [28]. White powder: Yield: 74%; mp: 126–128 °C (lit.: 130–131 °C) [28]. ^1^H NMR (400 MHz, CDCl_3_): *δ* = 5.29 (1H, br s, H-12), 4.04 (2H, t, *J =* 6.4 Hz, H-1’), 3.71–3.65 (1H, m, H-2), 3.43 (2H, t, *J =* 6.4 Hz, H-4’), 2.99 (1H, d, *J =* 10.0 Hz, H-3), 2.86 (1H, d, *J =* 11.0 Hz, H-18), 1.96–1.17 (24H, m, CH, CH_2_ in pentacyclic skeleton, H-2’, H-3’), 1.13, 1.02, 0.97, 0.93, 0.90, 0.81, 0.72 (3H each, all s, H-23–H-27, H-29 and H-30) ppm; ^13^C NMR (100 MHz, CDCl_3_): *δ* = 177.7 (C-28), 143.8 (C-13), 122.3 (C-12), 83.7 (C-3), 68.9 (C-2), 63.2 (C-1’), 55.3 (C-5), 47.5 (C-9), 46.7 (C-17), 46.4 (C-1), 45.8 (C-19), 41.7 (C-14), 41.3 (C-18), 39.4 (C-8), 39.3 (C-4), 38.2 (C-10), 33.8 (C-21), 33.1 (C-4’, C-29), 32.6 (C-7), 32.5 (C-22), 30.7 (C-20), 29.5 (C-30), 28.7 (C-15), 27.6 (C-23), 27.3 (C-2’), 25.9 (C-27), 23.6 (C-3’), 23.5 (C-11), 23.0 (C-16), 18.3 (C-6), 17.1 (C-26), 16.8 (C-24), 16.6 (C-25) ppm.

4-Bromobutyl-2α,3β-dihydroxy-urs-12-en-28-oate (**6**). Spectral data and all constants for that compound were as previously reported [28]. Yield: 66%; mp: 75–77 °C (lit.: 78–79 °C) [28]. ^1^H NMR (400 MHz, CDCl_3_): *δ* = 5.26 (1H, br s, H-12), 4.05–4.02 (2H, m, H-1’), 3.75–3.69 (1H, m, H-2), 3.46–3.40 (2H, m, H-4’), 3.03 (1H, d, *J =* 10.0 Hz, H-3), 2.24 (1H, d, *J =* 11.2 Hz, H-18), 2.04–0.86 (24H, m, CH, CH_2_ in pentacyclic skeleton, H-2’, H-3’), 1.27, 1.10, 1.05, 1.01, 0.96, 0.84, 0.76 (3H each, all s, H-23–H-27, H-29 and H-30) ppm; ^13^C NMR (100 MHz, CDCl_3_): *δ* = 177.5 (C-28), 138.3 (C-12), 125.3 (C-13), 83.9 (C-3), 69.0 (C-2), 63.2 (C-1’), 55.3 (C-5), 52.8 (C-18), 48.1 (C-17), 47.5 (C-9), 46.6 (C-1), 42.1 (C-14), 39.6 (C-8), 39.2 (C-19), 39.1 (C-20), 38.9 (C-4), 38.2 (C-10), 36.7 (C-22), 33.1 (C-4’), 32.9 (C-7), 30.7 (C-21), 29.5 (C-2’), 28.7 (C-23), 27.9 (C-15), 27.3 (C-3’), 24.2 (C-16), 23.6 (C-27), 23.4 (C-11), 21.2 (C-30), 18.3 (C-6), 17.2 (C-24), 17.0 (C-26), 16.8 (C-29), 16.7 (C-25) ppm.

#### 2.2.2. General Procedure for the Preparation of the Conjugates **1** and **2**

A mixture of the corresponding bromides **5** or **6** (1.0 mmol) and 3-[2-(4-pyridyl)vinyl]indole (1.0 mmol) in dry DMF (12 mL) was stirred at 85 °C in an argon atmosphere for 12 h. Then, the mixture was cooled to room temperature and evaporated under reduced pressure. The residue was purified by column chromatography on silica gel, using CH_2_Cl_2_/MeOH 30:1→10:1 to obtain pure compounds **1** or **2**. Yield 83–84%.

N-{4-[(2α,3β-dihydroxy-olean-12-en-28-oyl)-butyl]-(E)-4-[2-(1H-indol-3-yl)-vinyl]}pyridinium bromide (**1**). Dark orange powder. Yield: 83%; mp 206–208 °C (EtOH); [α]_D_^18^ +35 (*c* 0.02, CH_3_OH); IR (film) *ν*_max_ 1716 (C=O), 2920 (OH), 3370 (NH) cm^−1^; ^1^H NMR (500 MHz, MeOD/CDCl_3_): *δ* = 8.48 (2H, d, *J =* 6.4 Hz, H-5’, H-9’), 8.10 (1H, d, *J =* 16.0 Hz, H-10’ or H-11’), 8.00–7.97 (1H, m, H-15’), 7.88 (2H, d, *J =* 6.4 Hz, H-6’, H-8’), 7.80 (1H, s, H-12’), 7.46–7.44 (1H, m, H-18’), 7.24–7.22 (2H, m, H-16’, H-17’), 7.11 (1H, d, *J =* 16.0 Hz, H-10’ or H-11’), 5.21 (1H, br s, H-12), 4.43–4.40 (2H, m, H-4’), 4.12–3.99 (2H, m, H-1’), 3.53–3.47 (1H, d, *J* = 11.4, 4.4 Hz, H-2), 2.85–2.79 (2H, m, H-3, H-18), 2.00–0.72 (24H, m, CH, CH_2_ in pentacyclic skeleton, H-2’, H-3’), 1.09, 0.89, 0.89, 0.86, 0.86, 0.66, 0.60 (3H each, all s, H-23–H-27, H-29 and H-30) ppm; ^13^C NMR (125 MHz, MeOD/CDCl_3_): *δ* = 179.2 (C-28), 156.8 (C-7’), 144.7 (C-13), 143.6 (C-5’, C-9’), 139.1 (C-19’), 139.0 (C-10’ or C-11’), 133.8 (C-12’), 126.1 (C-14’), 124.3 (C-16’), 123.3 (C-12), 123.0 (C-8’, C-6’), 122.7 (C-17’), 121.3 (C-15’), 117.2 (C-10’ or C-11’), 115.3 (C-13’), 113.4 (C-18’), 84.2 (C-3), 69.2 (C-2), 64.2 (C-1’), 60.3 (C-4’), 56.2 (C-5), 48.5 (C-9), 47.8 (C-17), 47.5 (C-1), 46.7 (C-19), 42.7 (C-14), 42.4 (C-18), 40.3 (C-8), 40.1 (C-4), 38.9 (C-10), 34.6 (C-21), 33.6 (C-29), 33.5 (C-22, C-7), 31.4 (C-20), 29.1 (C-15), 29.0 (C-23), 28.5 (C-2’), 26.4 (C-27), 26.2 (C-3’), 24.4 (C-11), 24.1 (C-30), 23.9 (C-16), 19.3 (C-6), 17.8 (C-26), 17.4 (C-24), 17.2 (C-25) ppm; MS (HRMS): m/z [M-Br]^+^: 747.5182 C_49_H_67_N_2_O_7_ (calcd. 747.5095).

N-{4-[(2α,3β-dihydroxy-urs-12-en-28-oyl)-butyl]-(E)-4-[2-(1H-indol-3-yl)-vinyl]}pyridinium bromide (**2**). Orange powder. Yield: 84%; mp 219–221 °C (EtOH); [α]_D_^18^ +9.1 (*c* 0.03, CH_3_OH); IR (film) *ν*_max_ 1717 (C=O), 2921 (OH), 3370 (NH) cm^−1^; ^1^H NMR (500 MHz, MeOD): *δ* = 8.63 (2H, d, *J =* 6.0 Hz, H-5’, H-9’), 8.25 (1H, d, *J =* 16.0 Hz, H-10’ or H-11’), 8.09–8.06 (3H, m, H-6’, H-8’, H-15’), 7.90 (1H, s, H-12’), 7.50–7.49 (1H, m, H-18’), 7.29–7.26 (3H, m, H-10’ or H-11’, H-16’, H-17’), 5.21 (1H, br s, H-12), 4.52–4.49 (2H, m, H-4’), 4.13–4.01 (2H, m, H-1’), 3.54–3.50 (1H, dd, (*J* = 11.4, 4.4 Hz). H-2), 2.85 (1H, d, *J =* 10.0 Hz, H-3), 2.22 (1H, d, *J =* 11.0 Hz, H-18), 2.09–0.75 (24H, m, CH, CH_2_ in pentacyclic skeleton, H-2’, H-3’), 1.3, 1.10, 0.97, 0.91, 0.89, 0.67, 0.65 (3H each, all s, H-23–H-27, H-29 and H-30) ppm; ^13^C NMR (125 MHz, MeOD): *δ* = 179.2 (C-28), 157.5 (C-7’), 144.4 (C-5’, C-9’), 139.9 (C-19’), 139.7 (C-13), 139.2 (C-10’ or C-11’), 134.0 (C-12’), 127.0 (C-14’), 126.7 (C-12), 124.5 (C-16’), 123.4 (C-8’, C-6’), 122.9 (C-17’), 121.7 (C-15’), 117.8 (C-10’ or C-11’), 115.8 (C-13’), 113.6 (C-18’), 84.5 (C-3), 69.5 (C-2), 64.7 (C-1’), 60.7 (C-4’), 56.7 (C-5), 54.5 (C-18), 48.5 (C-17, C-9), 48.3 (C-1), 43.4 (C-14), 41.0 (C-8), 40.5 (C-19, C-20), 39.2 (C-4), 38.2 (C-10, C-22), 34.3 (C-7), 31.8 (C-21), 29.5 (C-2’), 29.3 (C-15), 29.2 (C-23), 26.5 (C-3’), 25.4 (C-16), 24.6 (C-11), 24.1 (C-27), 21.6 (C-30), 19.6 (C-6), 18.2 (C-26), 17.7 (C-24), 17.5 (C-29), 17.4 (C-25) ppm; MS (HRMS): m/z [M-Br]^+^: 747.5019 C_49_H_67_N_2_O_4_ (calcd. 747.5095).

### 2.3. Cell Culture

All cell lines used in this study (human non-small cell lung cancer—H1299 (Accession Number—CRL-5803), human lung carcinoma—A549 (Accession Number—CCL-185), human breast adenocarcinoma—MCF-7 (Accession Number—HTB-22), human breast ductal carcinoma—BT474 (Accession Number—HTB-20)) were purchased from American Type Culture Collection (ATCC, Manassas, VA, USA). Human primary dermal fibroblasts (HPDF) derived from a patch of skin of healthy donors (*n* = 3) were obtained from the biobank of the Institute for Regenerative Medicine, Lomonosov Moscow State University, collection ID: MSU_FB (https://human.depo.msu.ru (accessed on 14 September 2021)). Cells were grown in DMEM media supplemented with 10% fetal bovine serum, 100 μg/mL gentamycin, and 2 mM of glutamine at 37 °C in a 5% CO_2_ atmosphere.

### 2.4. Cytotoxic Assay of Conjugates

To carry out the MTT assay, 3500 cells per well were seeded in 96-well plates. A day after the seeding, triterpenoids or their derivatives dissolved in DMSO were added. DMSO was used as a control. At 48 h after cell seeding, 10 μL of 5 mg/mL Thiazolyl Blue (Paneko, Russia) solution was added to each well and cells were kept for 3 h at 37 °C in a CO_2_ incubator. After removing the thiazol-containing medium, 150 μL of isopropyl alcohol (supplemented with 40 mM HCl and 0.1% NP-40) was added to dissolve the MTT-formazan salt. The absorbance at 570 and 630 nm (reference) was measured using a BioRad iMark microplate reader (BioRad, Hercules, CA, USA). Four biological replicates were used for the experiment. The results were processed with Excel software (version number: 14.0.6023.1000 (32-bit)). IC_50_ values were calculated by using AAT Bioquest online calculator (https://www.aatbio.com/tools/ic50-calculator, accessed on 15 May 2023) and are represented as the mean ± SEM.

### 2.5. Oxidative Stress Assay

Oxidative stress was assessed after cell culture and incubation (as described above). For this, cells were harvested by trypsinization, washed with ice-cold PBS buffer (pH 7.4), and centrifuged (1000 rpm, 5 min). The resulting cells were resuspended in PBS. Cell population analysis for oxidative stress was performed on a Muse cell analyzer (Merck Millipore, Burlington, MA, USA) using the Muse Oxidative Stress Kit (MCH100111) according to the manufacturer’s protocol.

### 2.6. Isolation of Mitochondria from Rat Liver

Mitochondria were obtained from the liver of male Wistar rats (210–250 g) using the differential centrifugation method. The detailed procedure and composition of the isolation media were described earlier [29]. The suspension of isolated mitochondria contained about 60–70 mg of mitochondrial protein per ml (determined by the Bradford method).

### 2.7. Measurements of Mitochondrial Bioenergetics

The rate of O_2_ consumption by mitochondrial samples was estimated using Oxygraph Plus (Hansatech Instruments Ltd., Pentney, King’s Lynn, UK) [30]. The reaction medium included 200 mM sucrose, 20 mM KCl, 0.5 mM EGTA, 5 mM KH_2_PO_4_, and 10 mM Hepes/KOH, pH 7.4. The following combinations of substrates and reagents were used: 2.5 mM potassium glutamate + 2.5 mM potassium malate and 5 mM potassium succinate + 1 µM rotenone. Under these conditions, it is possible to assess the oxygen-consuming capacity of mitochondria through complexes I and II of the electron transport chain. To determine the rates of the phosphorylating respiration (State 3) and uncoupled respiration (State 3U_DNP_) of the mitochondria, 200 µM ADP and 50 µM 2,4-dinitrophenol (DNP) were added sequentially. The concentration of mitochondrial protein was 1 mg/mL.

The mitochondrial membrane potential (Δψ) was measured using a penetrating tetraphenylphosphonium bromide (TPP) cation and a TPP-sensitive electrode (Nico-Analyte Ltd., Moscow, Russia) as described previously [30]. The concentration of mitochondrial protein was 1 mg/mL.

The effect of compounds on the activity of the mitochondrial respiratory complexes (I, II, III, IV, I + III, II + III) was estimated using specific redox reactions according to the protocol using a Multiskan GO plate reader (Thermo Fisher Scientific, Waltham, MA, USA) [31,32]. The concentration of mitochondrial protein was 10 µg/mL.

The fluorescent probe Amplex Red (λ_ex_ = 560 nm; λ_em_ = 590 nm) and a plate reader Varioskan Lux were used to register mitochondrial H_2_O_2_ generation [30]. In this case, 0.15 mg of mitochondrial protein was incubated in the buffer containing 210 mM mannitol, 70 mM sucrose, 10 μM EGTA, 10 µM Amplex Red, 1 µM rotenone, 10 µM EGTA, horseradish peroxidase (HRP 1 a.u./mL), 10 mM Hepes/KOH, and at a pH of 7.4. Energization was achieved with 2.5 mM potassium glutamate + 2.5 mM potassium malate or 5 mM potassium succinate in the presence of 1 μM rotenone. The kinetics of H_2_O_2_ generation was recorded for three minutes.

### 2.8. Statistical Analysis

The data are expressed as the mean ± standard error of the mean (m ± SEM). GraphPad Prism 8 and Excel were used to analyze the data. The statistical significance of the differences between the means was evaluated using one-way analysis of variance (ANOVA) followed by Tukey’s post hoc test. The differences were considered statistically significant at *p* < 0.05.

## 3. Results

### 3.1. Chemistry

**Scheme 1:**
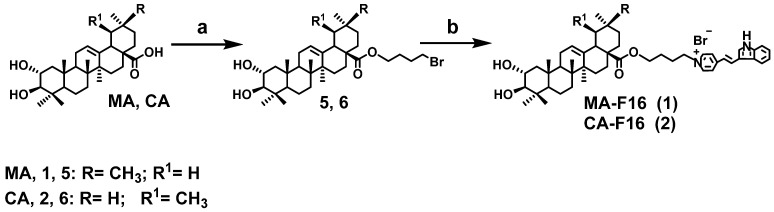
Synthesis conjugates with F16 **1** and **2**: Reagents and conditions: **a** 1,4-dibromobutane, K_2_CO_3_, CH_3_CN, DMF, 50 °C, 3 h; **b** (E)-4-(2-(1*H*-indol-3-yl)vinyl)pyridine*,* DMF, 85 °C, 12 h.

The starting compounds in the synthesis of F16 conjugates with maslinic and corosolic acids 1 and 2 were commercially available oleanolic and ursolic acids. These were transformed into maslinic and corosolic acids using the method described in [27] (Supplementary Material, Scheme S1). The five-step synthesis of these acids included sequential stages: benzyl protection of the carboxyl function in oleanolic and ursolic acids, oxidation of the 3-OH group in ring A using Jones reagent, treatment of the resulting 3-keto triterpenoids with m-chloroperoxybenzoic acid in the presence of catalytic amounts of sulfuric acid to yield the corresponding 2α-hydroxy ketones as sole products. The high stereoselectivity of the electrophilic attack of m- chloroperoxybenzoic acid on the C-2 atom of the formed enol intermediate can be explained by the rigid stereochemical control exerted by the β-oriented angular methyl group at the C-10 carbon atom of the triterpenic scaffold. Reduction of the 3-keto function in 2α-hydroxy ketones with NaBH_4_ resulted in a mixture of diastereoisomeric 2,3-dihydroxylated triterpenoids with predominant formation of 2*α*,3*β*-diols (2*α*,3*β*:2*α*,3*α*/4:1). Subsequent chromatographic separation of vicinal diols on a SiO_2_ column provided the target benzyl esters of maslinic and corosolic acids in an analytically pure form. The synthesis of maslinic and corosolic acids was completed by removing the benzyl protection of the triterpenic carboxyl function. The physicochemical and spectral characteristics of the semi-synthetic maslinic and corosolic acids corresponded to the literature data [27]. The obtained maslinic and corosolic acids were transformed into bromoalkyl esters by reaction with an excess of dibromobutane in the presence of potash and were conjugated with (E)-4-(1H-indol-3-ylvinyl)-pyridine in dimethylformamide at 85 °C [31]. As a result, MA-F16 (1) and CA-F16 (2) conjugates were obtained, and their structures were identified using IR, ^1^H NMR, ^13^C NMR, and MS spectra (Scheme 1). In the spectra of bromoalkyl esters 5 and 6 and conjugates MA-F16 (1) and CA-F16 (2), characteristic signals of protons in ring A of the triterpenic core at the 2α-OH function in the 3.53–3.47 ppm range appeared as a doublet of doublets (*J* = 11.4, 4.4 Hz). A strong NOESY correlation was observed between the protons of the C(10)-Me methyl group and H-2 indicated the configuration of the 2*α*-OH group. In the ^1^H NMR spectra, the presence of the (E)-4-(1H-indol-3- ylvinyl)pyridinium moiety is evidenced by two characteristic doublets for the pyridine ring at 8.09–7.88 and 8.63–8.48 ppm with *J* = 6.4 Hz, two vinyl group doublets at 7.1 and 8.1 ppm with *J* = 16 Hz, and three characteristic multiplets for the indole.

### 3.2. Survival of Different Tumor Cells Treated with Conjugates ***1*** and ***2***

**Figure 2:**
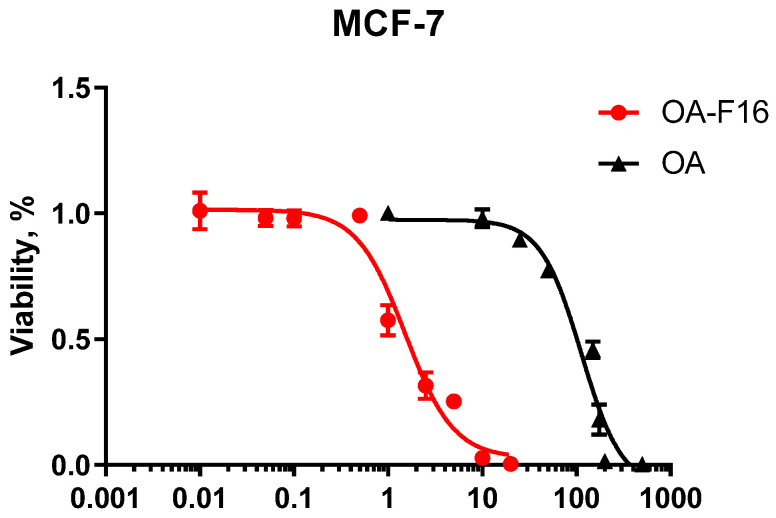
Dose dependence of the effect of OA, and OA–F16 on the survival of MCF-7 cells. Cytotoxicity was assessed 48 h after the addition of substances. Data (mean ± SEM) of 3 independent experiments are shown.

The resulting conjugates one and two were tested in vitro for their cytotoxic activity in four cancer cells H1299 and A549 (human non-small lung adenocarcinoma), breast cancer cell lines MCF-7 and BT474, and noncancer HPDF. In order to assess the effect of the hydroxyl group at the C-2 position of ring A in F16-derivatives of maslinic and corosolic acids on the antitumor activity, we also studied the cytotoxicity of conjugates of ursolic and oleanolic acids OA-F16 (3) and UA-F16 (4), synthesized by us earlier [21]. The parent compounds of ursolic and oleanolic acids were used as a standard. The screening results are presented in Table 1 and Figure 2.

All obtained compounds showed a significant synergistic enhancement of the antitumor effect compared to the original natural triterpenic acids. In particular, F16 conjugate with oleanolic acid OA-F16 (3) showed antitumor activity against A549, H1299, MCF-7, and BT474 cancer cells with IC_50_ values of 0.74, 0.93, 1.52, and 4.34 μM, while oleanolic acid showed activity with IC_50_s of 235.5, 139.3, 113.5, and 149.9 μM. The cytotoxicity of corosolic acid conjugate CA-F16 (2) against the A549 line was 36 times higher than that of ursolic acid (CA-F16 (2), IC_50_ = 1.9 μM; UA, IC_50_ = 68.9 μM). Contrary to our expectations, the presence of an additional hydroxyl function at the C-2 position of the triterpene core in the F16-conjugates of maslinic and corosolic acids resulted in a noticeable decrease in cytotoxicity compared to their structural analogues, F16-derivatives of oleanolic, and ursolic acids. Natural ursolic and oleanolic acids have not shown selectivity against tumor cells and human fibroblasts. The IC_50_ values of conjugates **1**–**4** for all tumor cells were low, but these compounds also lacked selectivity. Among the studied compounds, the highest cytotoxic activity against tumor cells H1299, A549 and MCF-7 was found for conjugate OA-F16. On the other hand, compound UA-F16 showed the highest difference in selectivity index between tumor cells H1299, A549 and MCF-7 and fibroblasts in the range (SI = 1.8–3.7). This is why conjugates OA-F16 and UA-F16 were chosen by us for further research. At the same time, the resistance of BT474 cells to all compounds was higher compared to fibroblasts.

**Figure 3:**
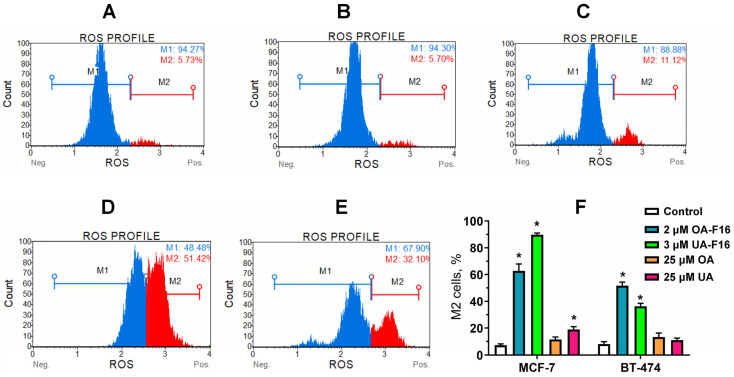
Typical distribution diagrams of BT474 (**A**–**E**) cell populations in the absence of additions (**A**) and in the presence of 25 μM OA (**B**), 25 μM UA (**C**), 2 μM OA-F16 (**D**) and 3 μM UA–F16 (**E**). (**F**) Calculation of ROS-positive MCF-7 and BT474 cell cells (%) in the absence (control) and the presence of OA, UA, OA–F16 and UA-F16. Means ± SEM are shown (*n* = 4–5). * Differences between control (without additions) and experiment (with tested compounds) were statistically significant (*p* < 0.05).

Previously, using several cell types and isolated rat liver mitochondria, we showed that the cytotoxic effect of F16 conjugates with lupane-type triterpenoids betulin and betulinic acid may be associated with their mitochondria-targeted, prooxidant action, and excessive production of ROS in the cell [31,33]. In this work, we tested the effect of triterpene acids and their most active conjugates (OA-F16 and UA-F16) on ROS production by breast cancer cell lines MCF-7 and BT474. Figure 3 shows typical flow cytometry data for the number of ROS-positive and ROS-negative cells in a culture of BT474 cells in the absence and presence of native oleanolic and ursolic acids and their conjugates with F16. One can see that native acids at a concentration of 25 μM do not significantly affect the production of ROS and, accordingly, the level of ROS-positive cells. At the same time, their conjugates at a significantly lower concentration (2 μM OA-F16 and 3 μM UA-F16, which have approximately the same cytotoxic effect) significantly increase the level of ROS-positive cells, which indicates a considerable amount of superoxide in the cells in the presence of these conjugates. The data on ROS production by breast cancer cell lines MCF-7 and BT474 in the presence of the studied compounds are summarized in Figure 3F and also show a significant increase in ROS production under the action of the conjugates.

### 3.3. Effect of Conjugates on the Functioning of Isolated Rat Liver Mitochondria

In this work, we also evaluated the effect of newly synthesized conjugates of oleanolic and ursolic acids on the functional activity of mitochondria. Isolated rat liver mitochondria were used as the object of study.

In the first step of this work, we evaluated the effect of native acids and their conjugates with F16 on the functional state of the organelles by the rate of mitochondrial respiration with glutamate/malate (substrates of complex I of the respiratory chain) or succinate (a substrate of complex II) in the presence of rotenone. Table 2 and Table 3 show that native acids at a concentration of 20 μM were able to increase the respiration rate of organelles in states 2 and 4, both in the presence of glutamate/malate and succinate as oxidation substrates. This was accompanied by a trend, or, in some cases (the effect of oleanolic acid on succinate-fueled respiration), a significant decrease in the respiratory control ratio, which reflects the degree of coupling of oxidation and phosphorylation in organelles. The conjugates, as expected, already showed their effect at a concentration of 10 μM and increased the rate of respiration in state 4, which was expressed in the case of organelle energization with succinate and was also accompanied by a significant decrease in the respiratory control ratio, and in the case of UA-F16, also by a decrease in the efficiency of ATP synthesis estimated as ADP/O ratio. An increase in the concentration of conjugates to 20 μM led to an increase in the rate of respiration in states 2 and 4 and, on the contrary, a decrease in the rate of ADP-stimulated respiration (state three) and respiration of organelles stimulated by the protonophore uncoupler 2,4-dinitrophenol (DNP, state 3U_DNP_). In this case, we also noted a significant decrease in the respiratory control and the ADP/O ratios.

**Figure 4:**
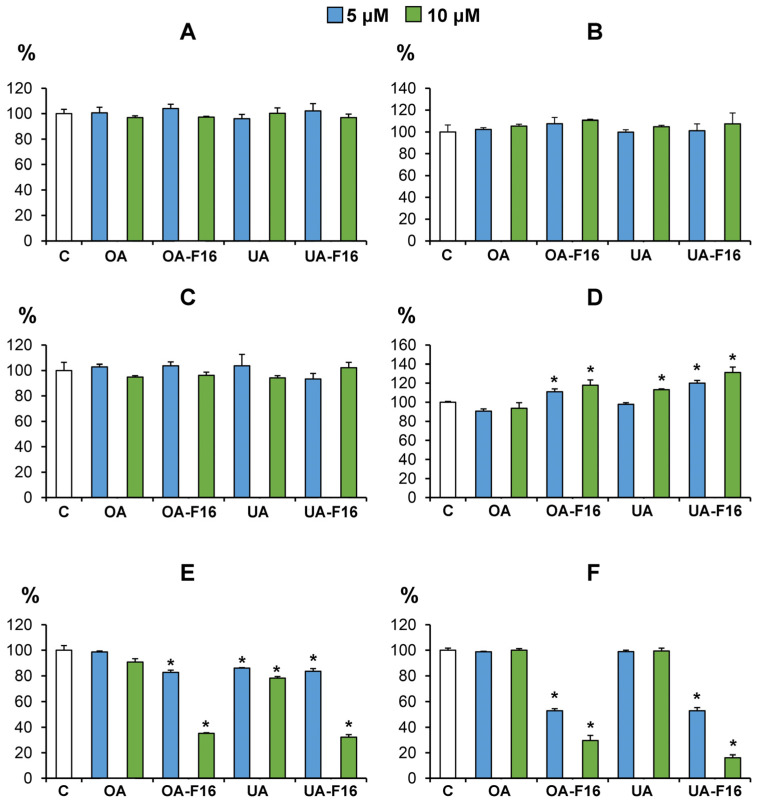
Effects of OA, UA and their F16 conjugates on the activity of complexes of the mitochondrial respiratory chain (values in % of activity compared with control). The concentration of mitochondrial protein was 0.01 mg/mL. In the absence of compounds (control), the activities of complexes I (**A**), II (**B**), III (**C**), IV (**D**), (I + III) (**E**) and (II + III) (**F**) were 399 ± 16, 484 ± 26, 657 ± 28, 469 ± 9, 702 ± 17 and 821 ± 11 nmol × min^−1^ × mg^−1^, respectively. The activity values in the absence of derivatives were taken as 100%. The results are presented as means ± SEM (*n* = 3). * Differences between control (C, without additions) and experiment (with tested compounds) were statistically significant (*p* < 0.05).

It is known that the effect of triterpenoids and their conjugates on the functioning of mitochondria may be due to the modulation of the activity of electron transport chain complexes and the mobility of its carriers [31,33,34]. One can see that native acids and their conjugates did not affect the activity of complexes I, II, and III. At the same time, the conjugates were able to significantly enhance the activity of cytochrome *c* oxidase (complex IV), and ursolic acid also showed this ability (Figure 4). On the other hand, we again noted the ability of F16 conjugates to exert a significant inhibitory effect on the combined activity of complexes I + III and II + III, which is thought to reflect the mobility of the hydrophobic carrier coenzyme Q between these protein ETC complexes. Such pronounced inhibitory effects of the conjugates already on the primary sites of the respiratory chain may apparently also contribute to a decrease in the intensity of ADP phosphorylation, which manifests itself in a decrease in the intensity of oxygen consumption by organelles in states 3 and 3U_DNP_.

**Figure 5:**
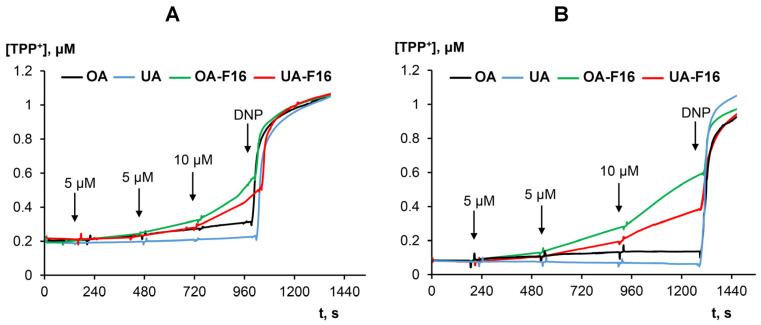
Effects of OA, UA, and their F16 conjugates on membrane potential of rat liver mitochondria. The incubation medium contained 210 mM mannitol, 70 mM sucrose, 1 mM KH_2_PO_4_, 10 μM EGTA, and 10 mM Hepes/KOH buffer at a pH of 7.4. 2.5 mM glutamate + 2.5 mM malate (**A**) or 5 mM succinate + 1 μM rotenone (**B**) were used as respiratory substrates. Other additions: 50 μM DNP. The concentration of mitochondrial protein was 1.0 mg/mL. The picture shows typical traces obtained on the same mitochondrial sample during the same experiment. Analogous results were obtained in two other independent experiments.

On the other hand, an increase in the rate of respiration in states 2 and 4 may be associated with the protonophore (uncoupling) activity of these compounds, which should be accompanied by a decrease in the membrane potential of the organelles. One can see that among native acids that only oleanolic acid was able to slightly reduce the membrane potential of organelles, estimated from the distribution of the tetraphenylphosphonium cation through the inner mitochondrial membrane (Figure 5). In this case, ursolic acid did not show such an ability. At the same time, F16 conjugates of these acids dose-dependently induced a decrease in the membrane potential of organelles, regardless of the substrate used. One should note that the OA-F16 conjugate was the most effective.

**Figure 6:**
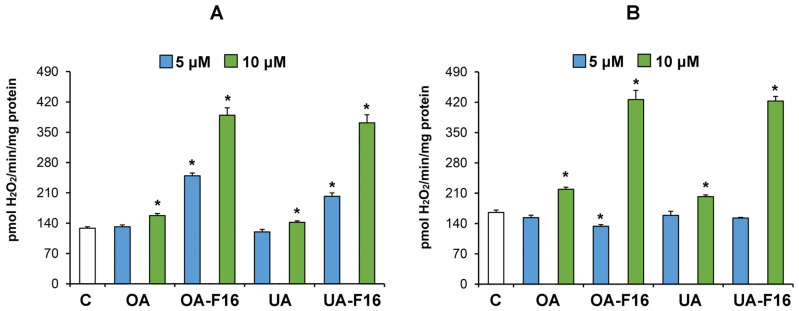
Effects of OA, UA, and their F16 conjugates on the rate of H_2_O_2_ production by rat liver mitochondria. The incubation medium contained 210 mM mannitol, 70 mM sucrose, 1 mM KH_2_PO_4_, 10 μM EGTA, and 10 mM Hepes/KOH buffer at a pH of 7.4. 2.5 mM glutamate + 2.5 mM malate (**A**) or 5 mM succinate + 1 μM rotenone (**B**) were used as respiratory substrates. The concentration of mitochondrial protein was 0.15 mg/mL. The results are presented as mean values ± SEM (*n* = 4). * Differences between control (C, without additions) and experiment (with tested compounds) were statistically significant (*p* < 0.05).

As shown above, the cytotoxic effect of the tested conjugates may be due to ROS hyperproduction (Figure 3). Mitochondria are one of the main sources of ROS in cells [35]. In this work, we examined the effect of native acids and their F16-conjugates on the rate of H_2_O_2_ production by rat liver mitochondria (Figure 6). One can see that at concentrations of 5–10 μM, all studied acids and F16 conjugates dose-dependently increased the rate of H_2_O_2_ production by mitochondria. However, the F16 conjugates of these acids had a much more pronounced prooxidant effect compared to the parental triterpenoids. One should note that we found an antioxidant effect for 5 μM of the OA-F16 compound, which manifests itself in a decrease in the production of H_2_O_2_ by organelles.

## 4. Discussion

Numerous secondary plant metabolites are known to have the potential for selective cytotoxic action against cancer cells. However, their effectiveness is seriously limited due to their high hydrophobicity, which limits the penetration of acids through the barriers of the body, primarily represented by lipid membranes. One of the approaches that can be applied to overcome this problem is the chemical modification of triterpene molecules by their conjugation with delocalized lipophilic cations (DLCs) and, in particular, F16. This DLC has the ability to pass through membranes and selectively accumulate in mitochondria due to the presence of a potential gradient across their inner membrane, which was confirmed in experiments on the visualization of the F16 conjugate and betulinic acid in cells [23].

In this work, we continue the development and testing of new derivatives of natural pentacyclic triterpene acids. We have synthesized F16 derivatives of well-known plant triterpene acids—oleanolic, maslinic, ursolic, and corosolic acids, natively demonstrating anticancer effects under in vitro conditions. One can see that for native acids, in particular, oleanolic and ursolic acids, the working concentrations that have a cytotoxic effect against cancer cells and healthy HPDF are approximately the same and reach tens and hundreds of micromoles, which seriously limits their therapeutic potential (Table 1). At the same time, the conjugation of these acids with F16 leads to a significant decrease in the active concentrations, down to the submicromolar scale, as is typical for the most active OA-F16 conjugate. In this case, we also noted a difference in the selectivity of this conjugate (SI = 1.3–2.8) between tumor cells and healthy fibroblasts. The highest selectivity was noted for the UA–F16 conjugate. At the same time, BT474 cells were the most resistant to all conjugates. Based on the results of this work and our previous data [23], we also calculated the Chou–Talalay combination index (CI), which allows evaluation of the synergism of the action of two substances, in particular, in a conjugated form. The CI quantitatively depicts synergism (CI < 1), additive effect (CI = 1), and antagonism (CI > 1) [36,37]. The CI for OA–F16 calculated for MCF7 cells based on the IC_50_ values for OA, F16 [23], and OA–F16 is 0.03, indicating a pronounced synergism. The CI in the case of UA–F16 is 0.05, which also indicates a synergistic effect.

As we have shown earlier [31,33], the cytotoxic effect of F16 conjugates and triterpenes may be based on ROS burst, leading to the development of oxidative stress and cell death. A similar picture was also found in the case of new conjugates. One can see that the most active OA-F16 and UA-A16 conjugates induce a significant increase in the level of ROS-positive cancer cells (MCF-7 and BT474), and in this case, they are superior to native acids in terms of efficiency (Figure 3).

Mitochondria are believed to be the source of ROS burst induced by F16 and triterpene conjugates [31,33]. In particular, the previous test is sensitive to superoxide, which is generated in mitochondria. Therefore, one could assume that mitochondria and the structures that ensure their correct functioning are the target of conjugates in cells. In order to clarify the nature of the observed processes, we evaluated the effect of the most active OA-F16 and UA-F16 conjugates on the functioning of classical objects: isolated rat liver mitochondria. We found that the tested conjugates induce organelle dysfunction, which manifests itself in a significant decrease in the efficiency of oxidative phosphorylation and ATP synthesis in mitochondria (Table 2 and Table 3), as well as a decrease in the membrane potential of organelles (Figure 5). In this case, they are superior to native OA and UA as inducers of mitochondrial dysfunction. This effect of the conjugates is apparently due to their membranotropic activity. Indeed, both tested conjugates are able to more effectively reduce the mitochondrial membrane potential and also modulate the activity of respiratory chain complexes of organelles localized in the inner membrane (Figure 4). In the latter case, we noted a significant reduction in the mobility of coenzyme Q, leading to a decrease in the efficiency of electron transport between complexes I and III, as well as complexes II and III of the ETC. One could assume that this also makes a significant contribution to the massive production of a large amount of ROS by mitochondria in the presence of conjugates (Figure 6). Indeed, the mobility of coenzyme Q is considered to play an important role in the regulation of ROS generation in the mitochondrial respiratory chain [35]. A similar effect of conjugates can also determine the ROS burst observed in cell cultures.

The data obtained in this work show that the pronounced cytotoxic effect of new F16 conjugates with triterpene acids (OA, UA, MA, and CA) may be associated with their significant effect on the functioning of mitochondria. These conjugates seem to cause dysfunction of mitochondria contributing to their active generation of ROS, the development of oxidative stress, and cell death in vitro. However, despite the mitochondrial targeting of the conjugates, the development of oxidative stress and mitochondrial dysfunction may not be the only reasons for the cytotoxic effect of the tested compounds. We can also mention the suppression of proliferation, which we observed earlier in the case of the conjugate of betulinic acid and the F16 cation [23] and a number of other effects of triterpene acids. At the same time, further search and synthesis of compounds that will have greater selectivity with respect to cancer cells is required.

## Figures and Tables

**Table 1:**
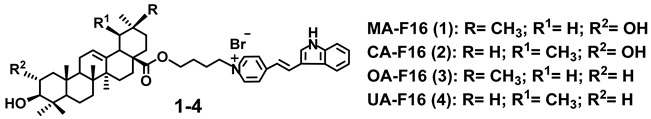
Cytotoxicity of new conjugates and natural triterpenoids: IC_50_ values MTT assays after 48 h of treatment are given in μM (three biological replicates were used for the experiment.

Compound	IC_50_ (μM)				
	H1299	A549	MCF7	BT474	HPDF
**OA-F16**	0.93 ± 0.07	0.74 ± 0.09	1.52 ± 0.16	4.34 ± 0.28	2.05 ± 0.58
**MA-F16**	10.50 ± 2.44	13.52 ± 2.02	7.44 ± 0.78	11.05 ± 2.51	9.30 ± 2.56
**UA-F16**	2.80 ± 0.25	2.40 ± 0.30	1.36 ± 0.74	5.18 ± 0.15	4.98 ± 0.9
**CA-F16**	4.01 ± 1.69	1.87 ± 0.14	1.89 ± 0.27	6.70 ± 0.35	1.15 ± 0.38
**OA**	139.33 ± 6.85	233.55 ± 22.05	113.5 ± 12.58	149.9 ± 25.12	156.3 ± 10.43
**UA**	97.60 ± 5.63	68.93 ± 19.63	40.6 ± 12.50	109.6 ± 19.08	59.07 ± 7.73

Data (mean ± SEM) of three independent experiments are shown.

**Table 2:** Effects of compounds on the respiration of rat liver mitochondria fueled by glutamate and malate.

Additions, μM	State 2	State 3	State 4	State 3U_DNP_	RC	ADP/O
	nmol O_2_/min/mg Protein				rel. un.	
-	7.03 ± 0.11	26.01 ± 0.46	10.35 ± 0.71	34.64 ± 0.25	2.55 ± 0.17	2.85 ± 0.08
Oleanolic acid						
20	8.39 ± 0.09 *	27.31 ± 0.31	13.23 ± 0.18 *	35.74 ± 0.66	2.11 ± 0.12	2.53 ± 0.12
OA-F16						
10	6.47 ± 0.27	23.82 ± 0.53 *	10.65 ± 0.24	31.34 ± 0.49 *	2.24 ± 0.11	2.80 ± 0.13
20	15.38 ± 0.30 *#	17.02 ± 0.97 *#	16.55 ± 0.54 *#	23.24 ± 0.61 *#^γ^	1.03 ± 0.04*#	0.64 ± 0.05 *#
Ursolic acid						
20	8.01 ± 0.15 *	26.92 ± 0.64	12.65 ± 0.32 *	36.66 ± 1.47	2.13 ± 0.09	2.82 ± 0.03
UA-F16						
10	7.70 ± 0.33	25.89 ± 0.86	11.78 ± 0.22	35.23 ± 0.89	2.20 ± 0.04	2.65 ± 0.05
20	14.75 ± 0.21 *#	19.35 ± 0.90 *#	19.06 ± 1.11 *#	28.29 ± 1.18 *#	1.02 ± 0.02 *#	0.74 ± 0.14 *#

Table shows means ± SEM (*n* = 4). RC = state 3/state 4. * *p* < 0.05 vs. control (no additions), # *p* < 0.05 vs. 20 µM of respective parental acids, ^γ^ *p* < 0.05 vs. 20 µM UA-F16.

**Table 3:** Effects of compounds on the respiration of rat liver mitochondria fueled by succinate.

Additions, μM	State 2	State 3	State 4	State 3U_DNP_	RC	ADP/O
	nmol O_2_/min/mg Protein				rel. un.	
-	12.62 ± 0.53	47.96 ± 0.59	14.00 ± 0.57	73.28 ± 2.44	3.44 ± 0.13	1.51 ± 0.06
Oleanolic acid						
20	15.06 ± 0.15 *	48.00 ± 0.60	17.18 ± 0.78 *	69.92 ± 0.68	2.80 ± 0.09 *	1.45 ± 0.05
OA-F16						
10	14.39 ± 0.65	48.46 ± 0.82	17.22 ± 0.93 *	69.57 ± 1.73	2.83 ± 0.20 *	1.41 ± 0.09
20	14.05 ± 0.18 *	41.57 ± 1.36 *#	33.23 ± 0.75 *#	56.46 ± 4.21 *#	1.25 ± 0.10 *#	1.15 ± 0.05 *#
Ursolic acid						
20	15.19 ± 0.27 *	49.15 ± 0.30	15.54 ± 0.62	75.91 ± 1.74	3.32 ± 0.12	1.43 ± 0.04
UA-F16						
10	14.42 ± 0.24 *	46.39 ± 0.30	17.51 ± 0.23 *	67.34 ± 2.12	2.65 ± 0.07 *	1.28 ± 0.04 *
20	15.86 ± 0.40 *	43.98 ± 1.31 *#	32.20 ± 1.68 *#	64.18 ± 3.81 #	1.37 ± 0.11 *#	0.86 ± 0.05 *#^γ^

Table shows means ± SEM (*n* = 4). RC = state 3/state 4. * *p* < 0.05 vs. control (no additions), # *p* < 0.05 vs. 20 µM of respective parental acids, ^γ^ *p* < 0.05 vs. 20 µM UA-F16.

## Data Availability

The data presented in this study are available upon request from the corresponding author.

## Supplementary Materials

The following supporting information can be downloaded at: https://www.mdpi.com/article/10.3390/membranes13060563/s1, Scheme S1. Synthesis of maslinic and corosolic acids.
