# Supplementary material for: Conjugation of Triterpenic Acids of Ursane and Oleanane Types with Mitochondria-Targeting Cation F16 Synergistically Enhanced Their Cytotoxicity against Tumor Cells
Source: Membranes (Basel). 2023 May 30;13(6):563. doi: 10.3390/membranes13060563 (PMC10303159; doi:10.3390/membranes13060563)

membranes

Supplementary Material: Conjugation of Triterpenic Acids of Ursane and Oleanane Types with Mitochondria-targeting Cation F16 Synergistically Enhanced Their Cytotoxicity Against Tumor Cells

Mikhail V. Dubinin, Darya A. Nedopekina, Anna I. Ilzorkina, Alena A. Semenova, Vyacheslav A. Sharapov, Eldar V. Davletshin, Natalia V. Mikina, Yuri P. Belsky, Anna Yu. Spivak, Vladimir S. Akatov, Natalia V. Belosludtseva, Jiankang Liu and Konstantin N. Belosludtsev

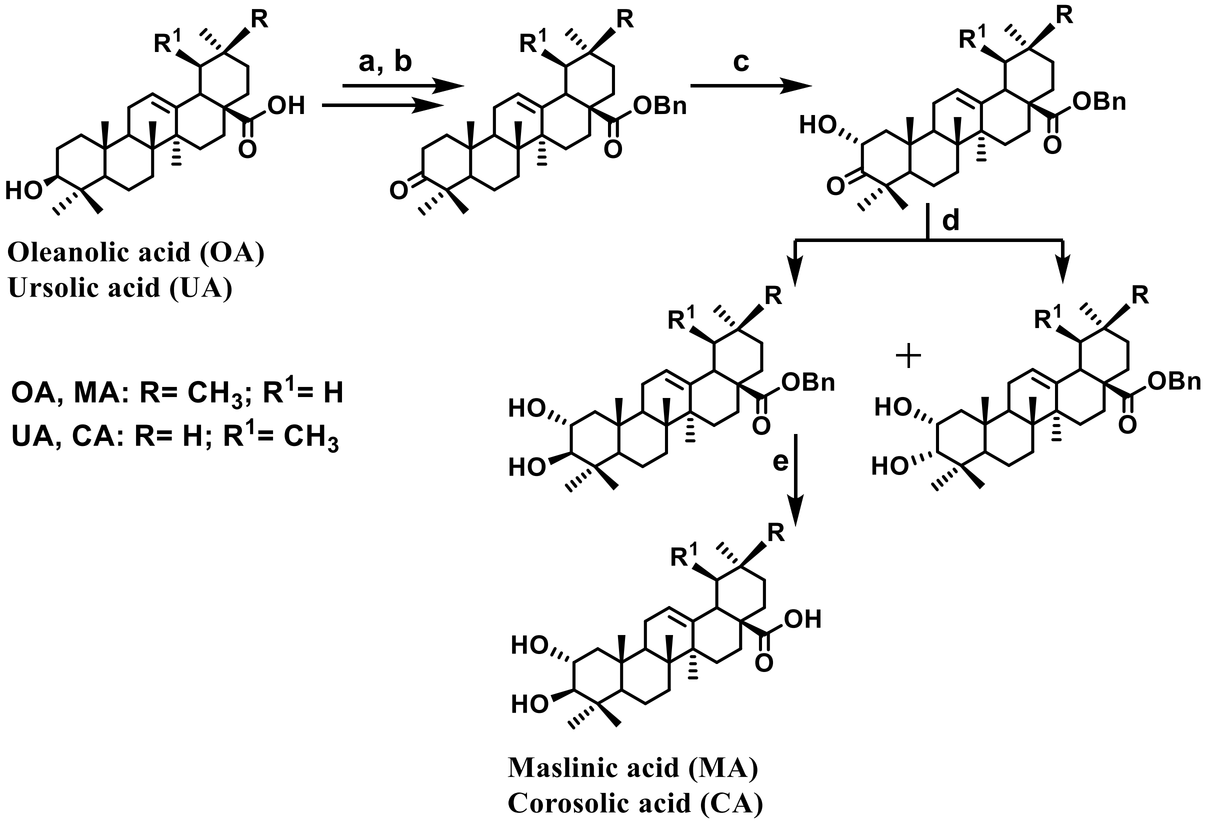

Scheme S1. Synthesis of maslinic and corosolic acids: Reagents and conditions: **a** BnCl, K_2CO_3, DMF, 50 °C, 2 h; **b** CrO_3, H_2SO_4, acetone, 0 °C; **c** *m*CPBA, H_2SO_4, MeOH–CH_2Cl_2, 0 °C; **d** $NaBH_4$ or $NaBH_4$-$CeCl_3$·$7H_2O$, MeOH–THF, rt; **e** Pd-C/10%, H_2, MeOH–THF, rt.

4-Bromobutyl-2α,3β-dihydroxy-olean-12-en-28-oate (5).

^{13}C NMR spectra (CDCl$_3$)

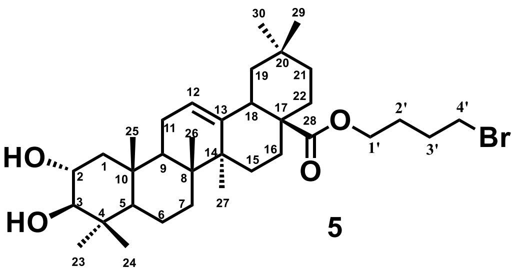

5

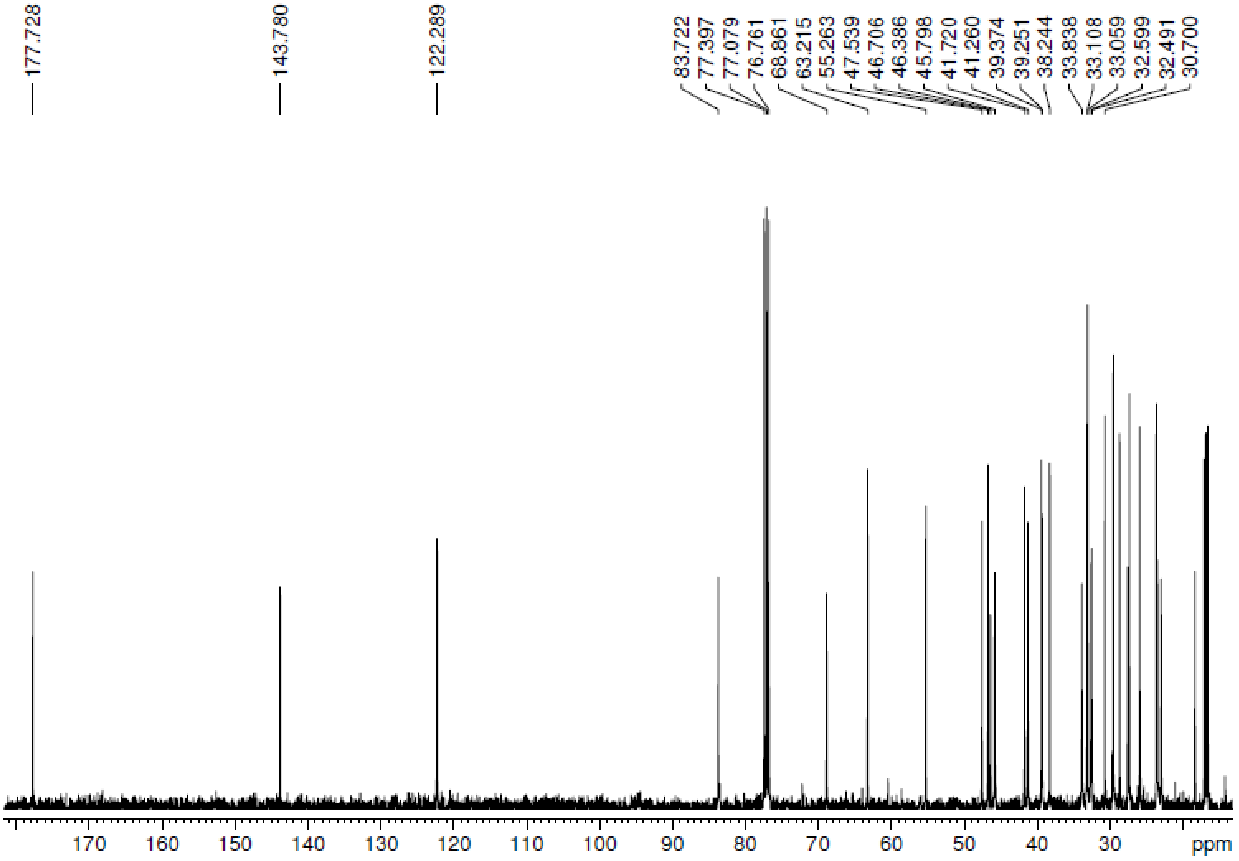

4-Bromobutyl-2α,3β-dihydroxy-olean-12-en-28-oate (5).

^{1}H NMR spectra (CDCl3)

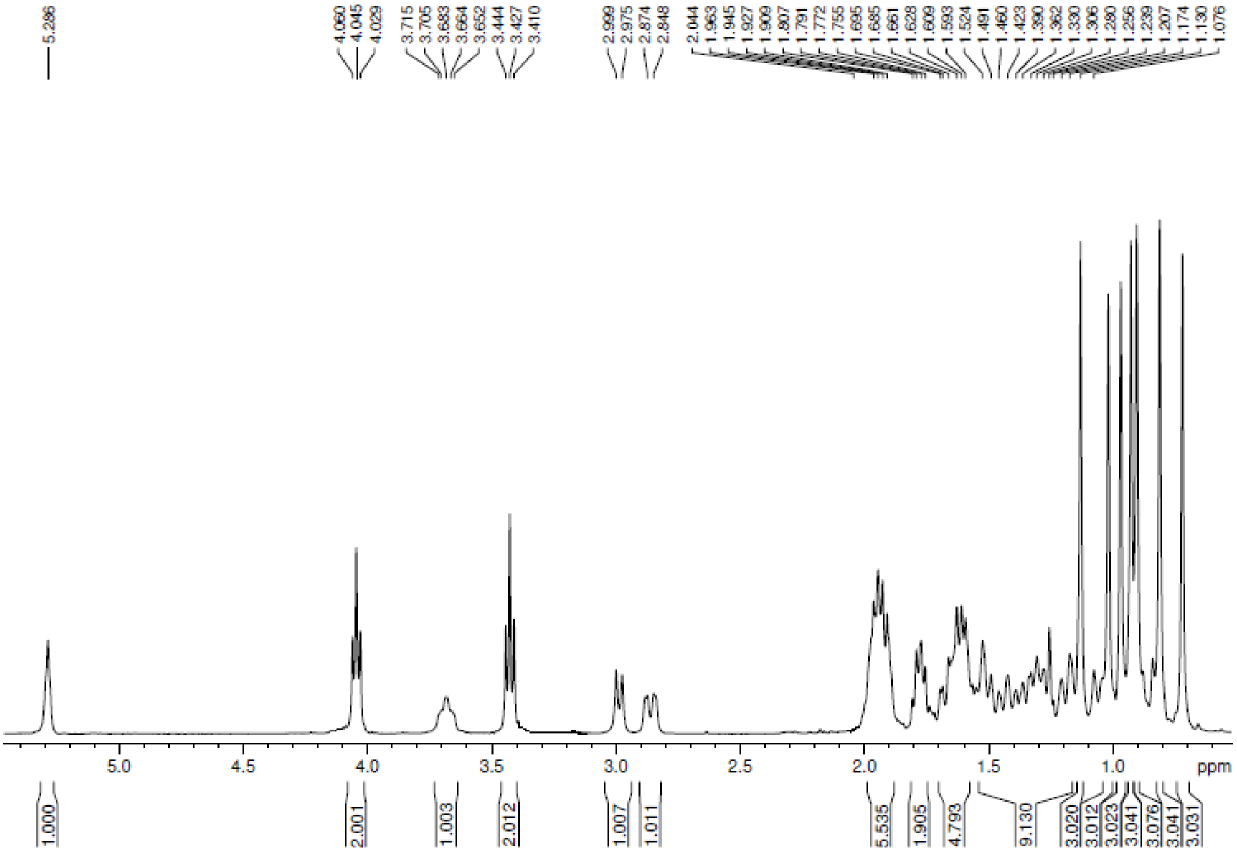

N-{4-[(2α,3β-dihydroxy-olean-12-en-28-oyl)-butyl]-(E)-4-[2-(1H-indol-3-yl)-vinyl]}pyridinium bromide (1). ^{13}C NMR spectra (MeOD/CDCl$_3$)

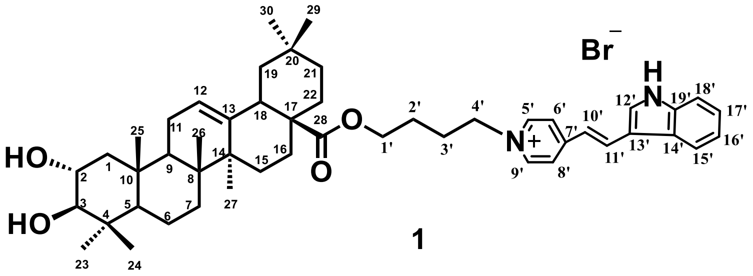

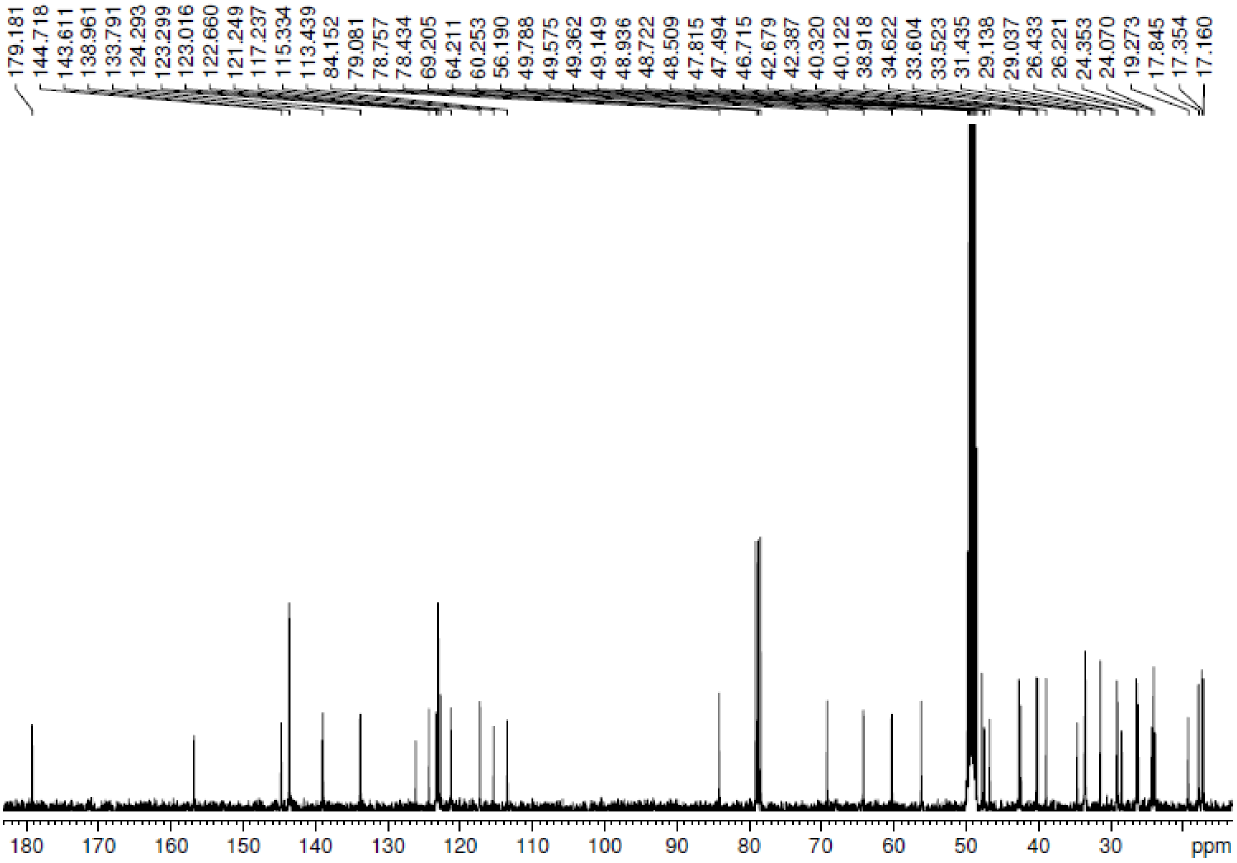

N-{4-[(2α,3β-dihydroxy-olean-12-en-28-oyl)-butyl]-(E)-4-[2-(1H-indol-3-yl)-vinyl]}pyridinium bromide (1). ^{1}H NMR spectra (MeOD/CDCl$_3$)

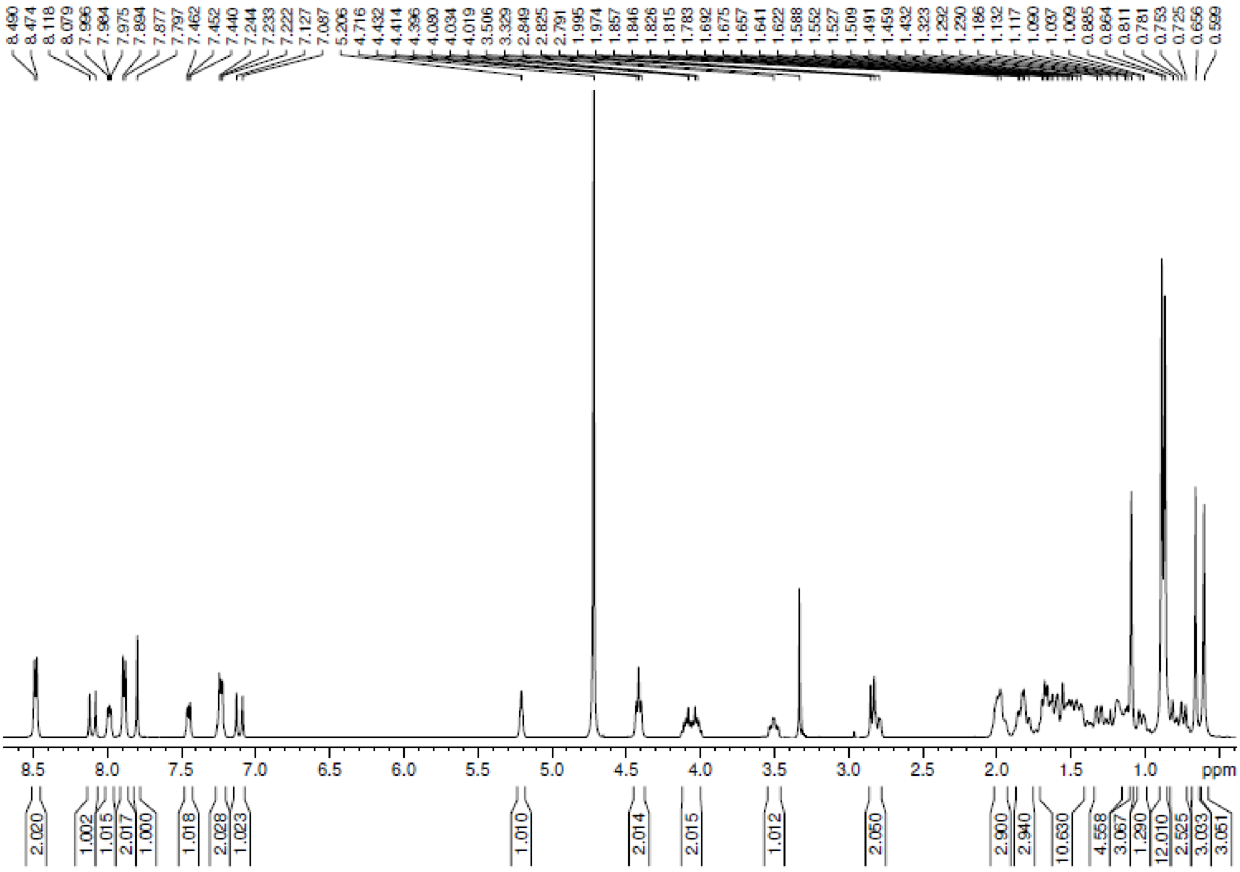

4-Bromobutyl-2α,3β-dihydroxy-urs-12-en-28-oate (6).

^{13}C NMR spectra (CDCl$_3$)

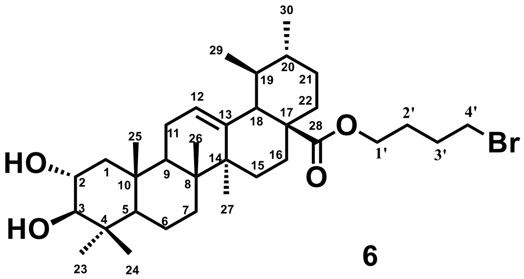

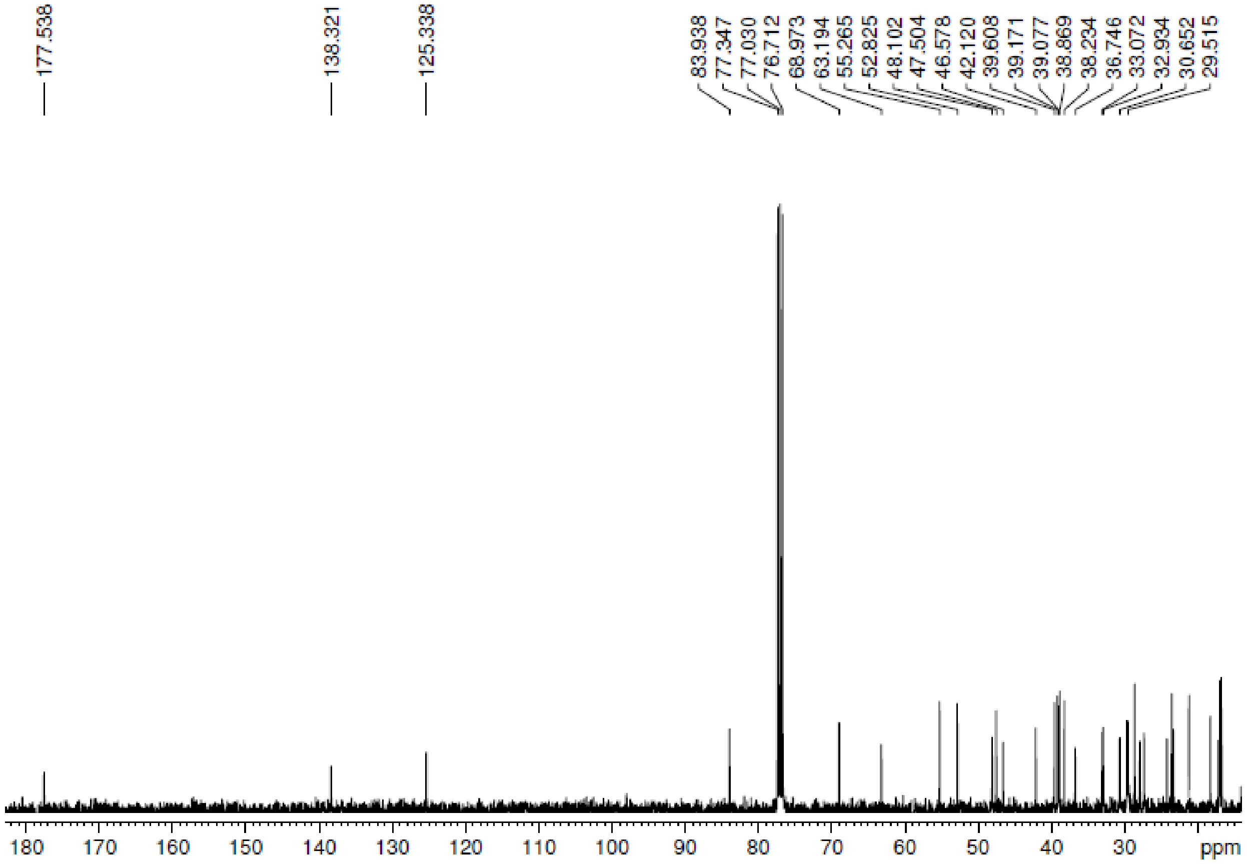

4-Bromobutyl-2α,3β-dihydroxy-urs-12-en-28-oate (6).

^{1}H NMR spectra (CDCl3)

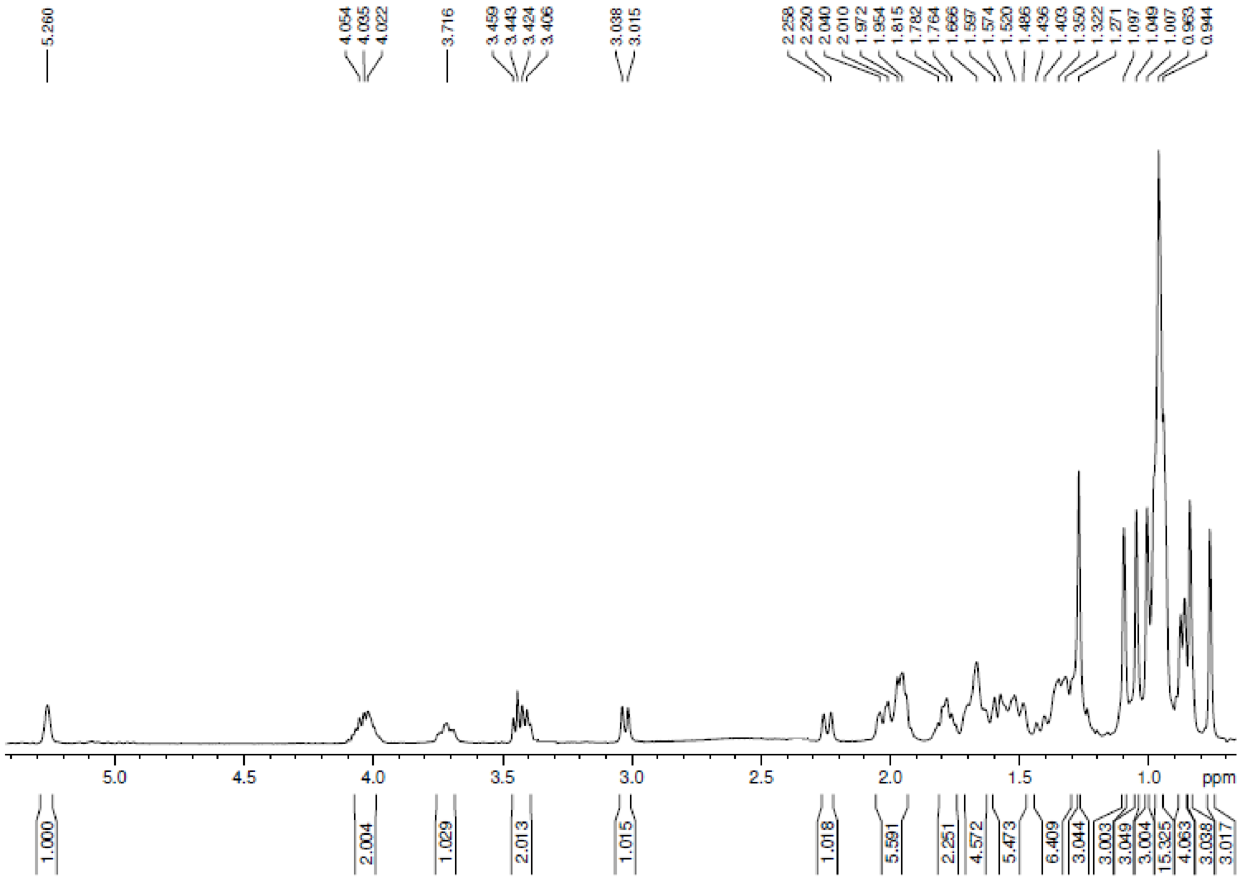

N-{4-[(2α,3β-dihydroxy-urs-12-en-28-oyl)-butyl]-(E)-4-[2-(1H-indol-3-yl)-vinyl]}pyridinium bromide (2). ^{13}**C NMR spectra (MeOD)**

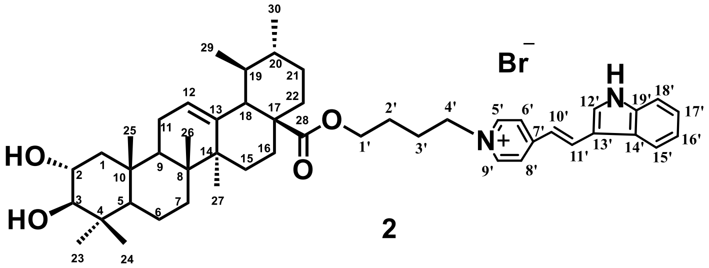

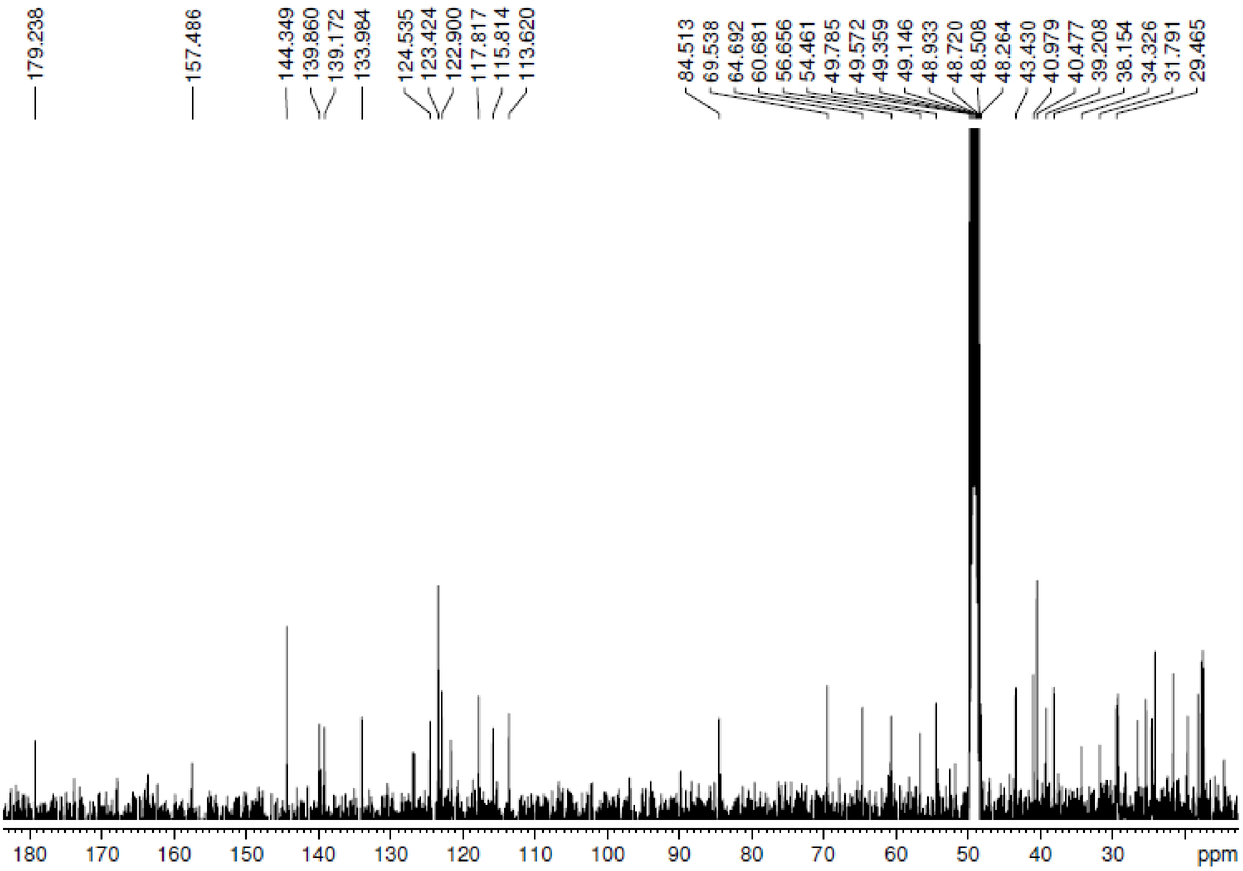

N-{4-[(2α,3β-dihydroxy-urs-12-en-28-oyl)-butyl]-(E)-4-[2-(1H-indol-3-yl)-vinyl]}pyridinium bromide (2). ^{1}H NMR spectra (MeOD)

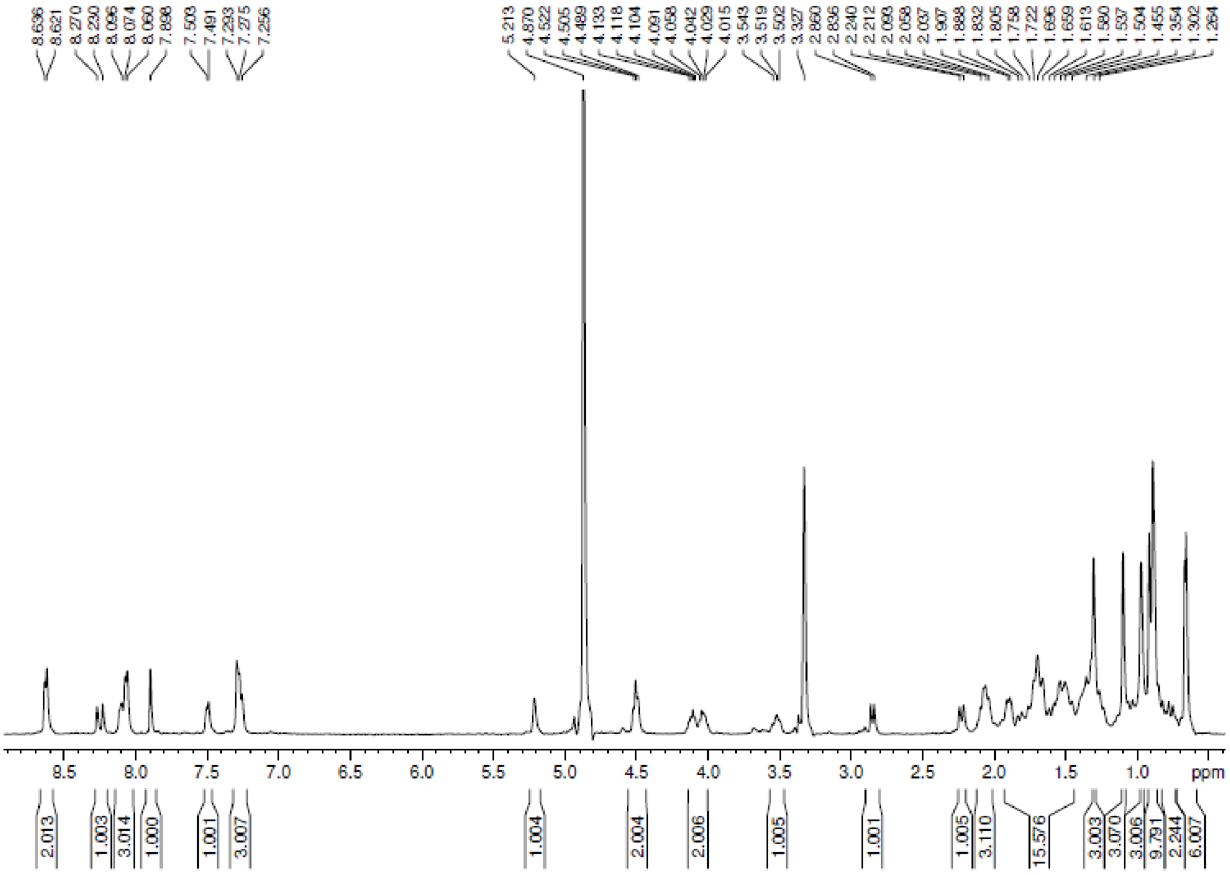

Supplement: Supplementary file 1 [file membranes-13-00563-s001.zip › membranes-2426906-supplementary.pdf]
